# The Anti-helminthic Compound Mebendazole Has Multiple Antifungal Effects against *Cryptococcus neoformans*

**DOI:** 10.3389/fmicb.2017.00535

**Published:** 2017-03-28

**Authors:** Luna S. Joffe, Rafael Schneider, William Lopes, Renata Azevedo, Charley C. Staats, Lívia Kmetzsch, Augusto Schrank, Maurizio Del Poeta, Marilene H. Vainstein, Marcio L. Rodrigues

**Affiliations:** ^1^Laboratório de Biologia Celular de Leveduras Patogênicas, Instituto de Microbiologia Paulo de Góes, Universidade Federal do Rio de JaneiroRio de Janeiro, Brazil; ^2^Centro de Biotecnologia, Universidade Federal do Rio Grande do SulPorto Alegre, Brazil; ^3^Fundação Oswaldo Cruz – Fiocruz, Centro de Desenvolvimento Tecnológico em SaúdeRio de Janeiro, Brazil; ^4^Departamento de Biologia Molecular e Biotecnologia, Universidade Federal do Rio Grande do SulPorto Alegre, Brazil; ^5^Veterans Administration Medical Center, NorthportNY, USA; ^6^Department of Molecular Genetics and Microbiology, Stony Brook University, Stony BrookNY, USA

**Keywords:** *Cryptococcus neoformans*, benzimidazoles, mebendazole, antifungal, biofilm, antifungal targets, macrophages

## Abstract

*Cryptococcus neoformans* is the most lethal pathogen of the central nervous system. The gold standard treatment of cryptococcosis, a combination of amphotericin B with 5-fluorocytosine, involves broad toxicity, high costs, low efficacy, and limited worldwide availability. Although the need for new antifungals is clear, drug research and development (R&D) is costly and time-consuming. Thus, drug repurposing is an alternative to R&D and to the currently available tools for treating fungal diseases. Here we screened a collection of compounds approved for use in humans seeking for those with anti-cryptococcal activity. We found that benzimidazoles consist of a broad class of chemicals inhibiting *C. neoformans* growth. Mebendazole and fenbendazole were the most efficient antifungals showing *in vitro* fungicidal activity. Since previous studies showed that mebendazole reaches the brain in biologically active concentrations, this compound was selected for further studies. Mebendazole showed antifungal activity against phagocytized *C. neoformans*, affected cryptococcal biofilms profoundly and caused marked morphological alterations in *C. neoformans*, including reduction of capsular dimensions. Amphotericin B and mebendazole had additive anti-cryptococcal effects. Mebendazole was also active against the *C. neoformans* sibling species, *C. gattii*. To further characterize the effects of the drug a random *C. gattii* mutant library was screened and indicated that the antifungal activity of mebendazole requires previously unknown cryptococcal targets. Our results indicate that mebendazole is as a promising prototype for the future development of anti-cryptococcal drugs.

## Introduction

*Cryptococcus neoformans* is a yeast-like pathogen that causes expressive brain damage in immunosuppressed individuals (Colombo and Rodrigues, 2015). The fungus reaches the lungs of humans after inhalation of environmental cells. In the immunosuppressed host, *C. neoformans* efficiently disseminates to the brain and causes meningitis (Kwon-Chung et al., 2014). Cryptococcal meningitis is a global problem resulting in thousands of deaths annually (Park et al., 2009). Most cases occur among people with HIV/AIDS. Poor and late diagnosis, limited access to antifungals and drug resistance are directly associated to the high fatality rate of cryptococcosis, especially in developing countries (Rodrigues, 2016).

The standard antifungal regimen for cryptococcal meningitis is a combination of amphotericin B with 5-fluorocytosine (Krysan, 2015). Amphotericin B is nephrotoxic and is intravenously administered (Sloan et al., 2009; Micallef et al., 2015), which demands considerable medical infrastructure. A 15-day intravenous treatment with liposomal amphotericin B is estimated to cost from €10.000 to €20.000 in Europe (Ostermann et al., 2014) and 5-fluorocytosine is not widely available outside rich areas (Krysan, 2015). As an alternative, fluconazole is frequently used, although it is associated with poorer outcomes and relapses (Sloan et al., 2009). In South Africa, more than 60% of people with culture-positive relapsed disease had fluconazole resistance (Govender et al., 2011). Hence, the need for new anticryptococcal therapies is clear. In this context, a new class of antifungals targeting the synthesis of fungal sphingolipids has been recently described, but its efficacy in humans is still unknown (Mor et al., 2015).

Drug repurposing has emerged as an alternative to the costly and time-consuming processes of drug discovery and development (Nosengo, 2016). In the field of antifungal development, sertraline, an anti-depressive agent, has been reported to be an *in vitro* and *in vivo* fungicidal compound that, in combination with amphotericin B, improves the outcome of cryptococcosis (Zhai et al., 2012; Rhein et al., 2016). Sertraline is now under phase III trial to determine whether adjunctive therapy will lead to improved survival (ClinicalTrials.gov, 2016).

In this manuscript, we aimed at finding anti-cryptococcal activity in a collection of drugs previously approved for use in human diseases. Our results are in agreement with the notion that benzimidazole-like compounds are interesting prototypes for the future development of efficient anti-cryptococcal agents interfering with fungal morphology, biofilm formation, cellular proliferation and intracellular parasitism. This study also supports the hypothesis that the antifungal activity of mebendazole might involve previously unknown cellular targets.

## Materials and Methods

### Strains and Growth Conditions

Strains H99 of *C. neoformans* (sorotype A) and R265 of *C. gattii* (sorotype B) were maintained in Sabouraud’s agar. For capsule size determination and fluorescence microscopy, fungal cells were cultivated in a minimal medium composed of glucose (15 mM), MgSO_4_ (10 mM), KH_2_PO_4_ (29.4 mM), glycine (13 mM), and thiamine-HCl (3 μM), pH5.5 for 48 h at 37°C with shaking. The *C. gattii* mutant library was maintained in 96-well plates containing yeast peptone-dextrose (YPD) broth with 30% glycerol at -20°C. The cell line J774.16 (murine macrophages) was maintained in Dulbecco’s Modified Eagle Medium (DMEM) supplemented with 10% of Fetal Bovine Serum (FBS), and 1% of penicillin-streptomycin at 37°C in 5% CO_2_ atmosphere_._ After four passages in culture medium, the macrophages were plated into 96 well plates for tests of mebendazole intracellular activity.

### Screening for Antifungal Activity in a Compound Collection

The National Institutes of Health (NIH) clinical collection (NCC) was screened for antifungal activity against *C. neoformans*. The NCC consists in a small molecule repository of 727 compounds arrayed in 96-well plates at 10 mM solution in DMSO. These compounds are part of screening library for the NIH Roadmap Molecular Libraries Screening Centers Network (MLSCN) and correspond to a collection of chemically diverse compounds that have been in phase I-III clinical trials. Each compound was first diluted to 1 mM in DMSO and stored at -20°C until use. For initial screening, all compounds were used at 10 μM in 100 μl of RPMI 1640 (two times concentrated) medium buffered with morpholinepropanesulfonic acid (MOPS) at pH 7 containing 2% of glucose, in 96-well plates. Final concentration of DMSO in all samples corresponded to 1%. *C. neoformans* cells (10^4^) suspended in 100 μl of water were added to each well. The plates were incubated at 37°C with shaking for 48 h. The optical density at 540 nm (OD_540_) was recorded using the FilterMax 5 microplate reader (Molecular Devices, Sunnyvale, CA, USA). Compounds producing values of OD_540_ smaller than 0.05 after fungal growth were selected for further studies. As further detailed in the section “Results,” mebendazole was the NCC compound selected for most of the analysis performed in this study.

### Analysis of Antifungal Activity of NCC Compounds

Values of minimum inhibitory concentrations (MICs) were determined using the methods proposed by the European Committee on Antimicrobial Susceptibility Testing (EUCAST) with minor modifications. NCC compounds showing antifungal activity were serially diluted (20 to 0.03 μM) in RPMI 1640 (two times concentrated, pH 7; 2% glucose) buffered with MOPS in 96-well plates. The inocula of *C. neoformans* (strain H99) and *C. gattii* (strain R265) were prepared following the EUCAST protocol (Subcommittee on Antifungal Susceptibility Testing of the EECfAST, 2008; Arendrup et al., 2012). The plates were incubated at 37°C with shaking for 48 h. MIC values corresponded to the lowest compound concentration producing inhibition of fungal growth. For determination of fungicidal activity, *C. neoformans* cells were grown overnight in YPD broth at 30°C, washed in PBS and suspended in RPMI 1640 buffered with MOPS, pH 7. The yeast suspension was adjusted to 2 × 10^4^ cells per 10 ml of RPMI 1640 (pH 7; 2% glucose) buffered with MOPS and supplemented with 1.25, 0.3125, and 0.078 μM of antifungal compounds. Final concentration of DMSO corresponded to 0.6% in all samples. The samples were then incubated at 37°C in a rotary shaker at 200 rpm. Aliquots were taken at different time points and plated onto YPD agar plates that were incubated at 37°C for 48 h. The numbers of CFU were then counted and recorded. The minimum fungicidal concentration (MFC) was defined as the lowest drug concentration inhibiting CFU formation in at least 90% in comparison to systems containing no antifungals.

### Antifungal Activity of Mebendazole against Intracellular Cryptococci

To assess antifungal activity against intracellular *C. neoformans*, the fungus was first opsonized by incubation (20 min, 37°C, with shaking) in DMEM containing 10% FBS and 10 μg/ml of the 18B7 IgG1, an opsonic monoclonal antibody to GXM (Casadevall et al., 1998) kindly donated by Dr. Arturo Casadevall (Johns Hopkins University). The fungus was washed with fresh DMEM and incubated with J774.16 macrophages (1:1 ratio, 5 × 10^5^ cells/well of 96 well-plates) for 2 h in DMEM containing 10% FBS, 0.3 μg/ml LPS and 0.005 μg/ml IFNγ (37°C, 5% CO_2_). After interaction of *C. neoformans* with macrophages, the systems were washed with DMEM to remove extracellular fungal cells and fresh DMEM containing 10% FBS and variable concentrations of mebendazole (0.25, 0.5, and 1 μM, 200 μl/well) was added to each well. The plates were incubated at 37°C with 5% CO_2_. After 8 or 24 h, supernatants were collected for inoculation of YPD agar plates and subsequent CFU counting. Alternatively, infected macrophages were lysed with cold, distillated water and the resulting lysates were plated onto YPD agar plates for CFU counting. To evaluate the toxicity of mebendazole for J774.16 macrophages, 5 × 10^5^ macrophages suspended in DMEM containing 10% FBS were plated in each well of 96 wells plates and incubated overnight at 37°C with 5% CO_2_. The medium was supplemented with fresh DMEM containing 10% FBS and mebendazole (0.25, 0.5, and 1 μM) or 0.5% DMSO. The plate was incubated at 37°C in a 5% CO_2_ atmosphere. After 48 h, the systems were washed with DMEM and 50 μl of 3-(4,5-dimethylthiazol-2-yl)-2,5-diphenyltetrazolium bromide (MTT) at 5 mg/ml was added to each well, for further incubation for 4 h (37°C, 5% CO_2_) under light protection (Mosmann, 1983). Supernatants were removed and 200 μl DMSO was added for dissolution of formazan crystals. Absorbances were recorded using the FilterMax 5 microplate reader (Molecular Devices, Sunnyvale, CA, USA) at 570 nm.

### Analysis of Synergistic Effects

Synergistic activity between mebendazole and standard antifungal drugs was determined on the basis of the calculation of the fractional inhibitory index (FIC) (Mor et al., 2015). Briefly, mebendazole (denominated drug A) was serially diluted (0.38–0.006 μg/ml, 8 dilutions) in 96-well plates. Standard antifungals (denominated drugs B) were serially diluted (11 dilutions) from 16 to 0.015 μg/ml (amphotericin B) or 64 to 0.06 μg/ml (fluconazole). The FIC was defined as:

$$\frac{\text{MIC combined}}{\text{MIC drug A alone}}+\frac{\text{MIC combined}}{\text{MIC drug B alone}}$$

Synergism was categorized as follows: strongly synergistic effect, FIC < 0.5; synergistic effect, FIC < 1; additive effect, FIC = 1; no effect, 1 < FIC < 2; antagonistic effect, FIC > 2 (Mor et al., 2015).

### Effects of Mebendazole on Glucuronoxylomannan (GXM) Release

*Cryptococcus neoformans* cells (10^5^/well of 96-well plates, final volume of 200 μl, duplicates) were cultivated in RPMI 1640 buffered with MOPS, pH 7. The medium was supplemented with mebendazole (0.3125–0.001 μM). After 48 h of incubation at 37°C with shaking, the optical density of 540 nm (OD_540_) was recorded using the FilterMax 5 microplate reader (Molecular Devices, Sunnyvale, CA, USA). The plate was centrifuged for 10 min and supernatants were used for GXM quantification by ELISA using the protocol described by Casadevall et al. (1992). *C. neoformans* viability was monitored by propidium iodide (PI) staining of fungal cells. For this analysis, fungal cells obtained after exposure to 0.3125, 0.15625, and 0.078 μM mebendazole as described above were stained with 1 mg/ml PI for 5 min on ice and analyzed by flow cytometry in a FACS Cabibur (BD Biosciences, CA, USA). The percentage of stained (non-viable) cells was obtained with the FlowJo 7 software.

### Analysis of Capsular Size and Morphology

*Cryptococcus neoformans* was grown overnight in YPD broth and washed twice with PBS. The fungus was then suspended in minimal medium containing sub-inhibitory concentrations of mebendazole and incubated for 48 h at 37°C with shaking. *C. neoformans* cells were collected by centrifugation, washed in PBS and analyzed by microscopic approaches. For capsule size determination, the suspension was counterstained with India ink and placed onto glass slides. The suspensions were covered with glass coverslips and analyzed with an Axioplan 2 (Zeiss, Germany) microscope. Capsule size, calculated with the ImageJ Software, was defined as the distance between the cell wall and the outer border of the capsule. Cell diameters were determined using the same software. For additional analysis of capsular morphology, cellular suspensions were processed for fluorescence microscopy. Staining reagents used in this analysis-included calcofluor white (cell wall chitin, blue fluorescence) and the monoclonal antibody 18B7 (Casadevall et al., 1998). *C. neoformans* cells were prepared for fluorescence microscopy following the protocols established by our laboratory for routinely analysis of surface architecture (Rodrigues et al., 2008).

### Effects of Mebendazole on *C. neoformans* Biofilms

*Cryptococcus neoformans* was grown in Sabouraud’s dextrose broth for 24 h at 30°C with shaking. The cells were centrifuged at 3,000 *g* for 5 min, washed twice with PBS, suspended in minimal medium and adjusted to a density of 10^7^ cells/ml. Cell suspensions (100 μl) were added into quadruplicate wells of polystyrene 96-well plates (Greiner Bio-One, Australia), following incubation at 37°C for 48 h. The wells containing biofilms were washed three times with PBS to remove non-adhered cryptococcal cells. Fungal cells that remained attached to the wells were considered mature biofilms. To evaluate the susceptibility of *C. neoformans* biofilms to mebendazole, 100 μl solutions (31.25, 15.63, 3.13, 1.56, 0.31, and 0.16 μM) were added to each well. Amphotericin B and fluconazole (2 and 8 μg/ml, respectively) were used as control systems of antifungal activity. Negative controls corresponded to wells containing only water and untreated biofilms. Mature biofilms and antifungal drugs were incubated at 37°C for 24 h, washed three times with PBS and the biofilm metabolic activity quantified by the 2,3-bis (2-methoxy-4-nitro-5-sulfophenyl)-5-[(phenylamino) carbonyl]-2H-tetrazolium hydroxide (XTT) reduction assay (Meshulam et al., 1995). Prior studies demonstrated that the XTT reduction assay measurements correlate with biofilm and fungal cell number (Martinez and Casadevall, 2007). In addition to testing the effects of mebendazole on established biofilms, we evaluated whether this compound would inhibit biofilm formation. Cryptococcal cells were suspended in minimal medium and adjusted to a density of 10^7^ cells/ml in the presence or absence of mebendazole (31.25, 15.63, 3.13, 1.56, 0.31, and 0.16 μM). These suspensions were added in quadruplicates to the wells of polystyrene 96-well plates, following incubation at 37°C for 48 h. Amphotericin B and fluconazole (2 and 8 μg/ml, respectively) were used as antifungal controls. In negative controls, wells contained only ultrapure water. The wells were washed three times with PBS and biofilm formation was quantified by the XTT assay.

### Analysis of the Antifungal Activity of Mebendazole against a Collection of *C. gattii* Mutants

A collection of randomly generated *C. gattii* mutants was screened for identification of possible cellular targets for antifungal activity. Mutants (*n* = 7,569) were generated by insertional mutagenesis after incubation of *C. gattii* with *Agrobacterium tumefaciens* as previously described (Idnurm et al., 2004). All colonies that grew on YPD hygromycin plates were selected and maintained at -20°C in 96 wells plates containing 200 μl/well of YPD broth. Before exposure to mebendazole, mutant cells were first grown for 72 h (30°C) in 200 μl of YPD distributed into the wells of 96 wells plates. The antifungal activity of mebendazole against *C. neoformans* was reproduced in the *C. gattii* R265 strain. The mutants were tested for their ability to grow in the presence of RPMI 1640 supplemented with MOPS, 2% of glucose, 1% DMSO and 10 μM mebendazole. Resistance phenotypes (A_540_ > 0.3) were selected for dose-response tests of antifungal activity as described above and potential target identification was performed as detailed below.

### Identification of Potential Cellular Targets Required for the Antifungal Activity of Mebendazole

Based on clear resistant phenotypes, two *C. gattii* mutant strains were selected for target identification by polymerase chain reaction (PCR). The fungus was cultivated overnight in 10 ml YPD at 30°C with constant rotation. Each culture (2 ml) was centrifuged for 2 min at 4,000 × *g* and cell pellets were washed twice with PBS and collected for DNA extraction (Bolano et al., 2001). The cells were suspended in 400 μl of lysis buffer (50 mM Tris-HCl 1 mM EDTA, 200 mM NaCl, 2% Triton X100, 0.5% SDS, pH 7.5), for further addition of 1 volume of a phenol-chloroform mixture (pH 8) and 100 μl of 2-μm, acid washed glass beads. Mechanical disruption was performed by alternate 1-min cycles of vortexing and ice incubation. Lysates were centrifuged 4,000 × *g* for 20 min at 4°C. Supernatants were collected and DNA was ethanol-precipitated overnight at -20°C for subsequent treatment with RNAse (Bolano et al., 2001). Identification of missing genes in the mutants was performed using inverse PCR (Pavlopoulos, 2011). DNA was quantified using the Qubit reagent (Invitrogen) and 1 μg of each sample was cleaved separately with BglII, SalI, or StuI (Promega) restriction enzymes. The cleavage product was submitted to T4 DNA ligase reaction (New England) followed by PCR using primers for inverse PCR. Amplicons were gel-purified with the QIAquick Gel Extraction Kit (Qiagen). For the DNA sequencing reaction, 50 ng of each sample and 5 pmol of each primer were used. Sequences were obtained in an ABI-Prism 3500 Genetic Analyzer (Applied Biosystems) and their qualities were determined by a electropherogram analysis based on phred^1^. The identification of genes interrupted by the T-DNA was performed by comparison of each sequenced DNA fragment, which correspond to the T-DNA flanks, with the genomic sequence of *C. neoformans* H99 strain, available in the *Cryptococcus* genome databases (Broad Institute^2^) using BLASTn. Orthologs distribution was evaluated using the OrthoMCL database (Chen et al., 2006).

Mutants showing resistance to mebendazole were analyzed for their ability to produce melanin and extracellular GXM as previously described by our group (Rodrigues et al., 2015).

### Statistical Analyses

Statistics were obtained with the GraphPad Prism 6.0 software. Unpaired *t* student test was used for mebendazole toxicity analysis. The variance two-way ANOVA was carried out using Tukey’s and Bonferroni’s comparisons test for fungicidal activity and intracellular activity of mebendazole in macrophages. For biofilm analyses, one-way ANOVA was performed using Dunnett’s multiple comparisons.

## Results

### Selection of Mebendazole as a Potential Anti-cryptococcal Agent

**FIGURE 1:**
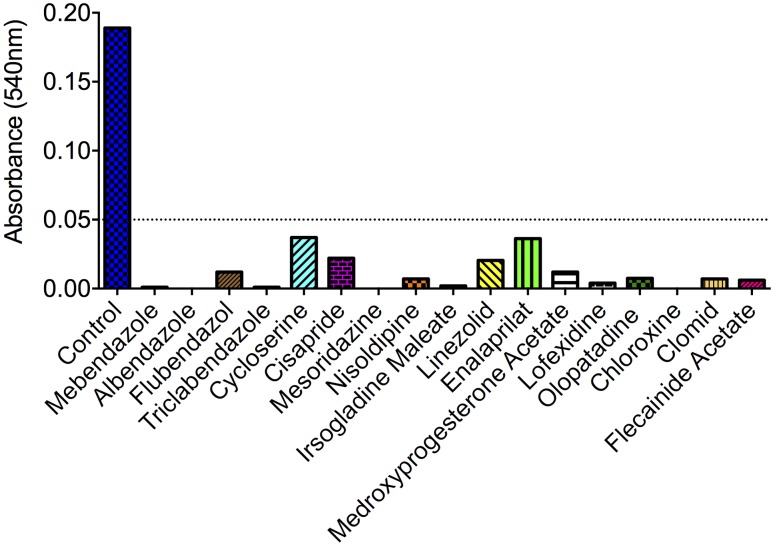
**Identification of small-molecule inhibitors of *Cryptococcus neoformans* via drug-repurposing screening.** Out of 727 compounds tested, 17 molecules induced *A*_540_ values smaller than 0.05 (dotted line). Compounds with no antifungal activity are not shown. For description of the primary activity of each molecule, see section “Results.” Data illustrate a representative experiment of two independent screenings.

Of the 727 drugs tested at 10 μM, 17 compounds were active against *C. neoformans*, including antibacterials (chloroxine, cycloserine, and linezolid), a neuroleptic drug (mezoridazine), calcium channel blockers (nisoldipine and enalaprilat), antiarrhythmic agents (flecainide acetate), drugs for gastrointestinal malignances (irsogladine maleate and cisapride), gynecologic regulators (medroxyprogesterone acetate and clomid), an anti-histaminic (olopatadine) and the anti-helminthic benzimidazoles (mebendazole, albendazole, flubendazole, and triclabendazole) (**Figure 1**). Noteworthy, none of the molecules showing antifungal activity were structurally related, except the benzimidazoles. Considering the currently observed efficacy in inhibiting the growth of *C. neoformans*, their structural similarity and previously described anti-cryptococcal activity (Cruz et al., 1994), we selected benzimidazoles for further investigation in our model.

**FIGURE 2:**
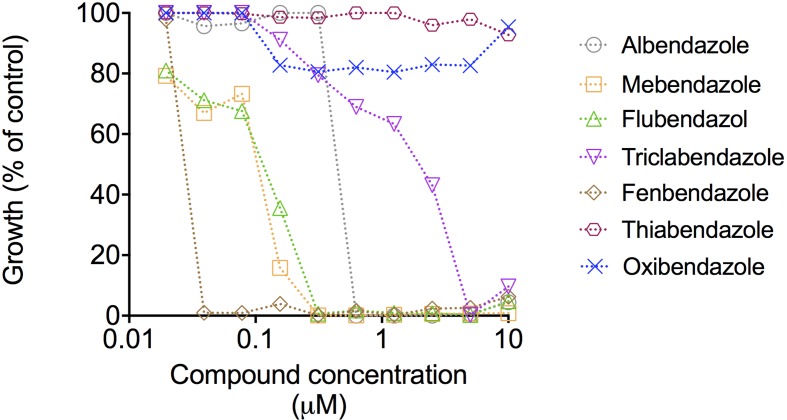
**Dose-response curves of the antifungal effects of benzimidazoles against *C. neoformans*.** Curves represent *C. neoformans* growth in comparison with vehicle-treated cells (DMSO). Data illustrate a representative experiment of three independent replicates.

We extended the results obtained with the compound collection to dose-response tests using the four benzimidazoles showing antifungal activity and other related molecules, including thiabendazole, oxibendazole, and fenbendazole. The most active compounds were mebendazole, fenbendazole, and flubendazole (**Figure 2**). Mebendazole and flubendazole produced the same MIC values (0.3125 μM). Fenbendazole was the most potent compound, with a MIC corresponding to 0.039 μM. Mebendazole, however, efficiently penetrates the brain in animal models (Bai et al., 2015) and is in clinical trial for the treatment of pediatric gliomas in humans (ClinicalTrials.gov, 2013). Considering that the worse clinical outcome of cryptococcosis includes colonization of the brain, we selected mebendazole as the molecular prototype for our subsequent analyses of fungicidal effects, ability to kill intracellular cryptococci, effects on fungal morphology, interference on fungal biofilms and identification of cellular targets.

### Mebendazole Is Fungicidal against *C. neoformans*

**FIGURE 3:**
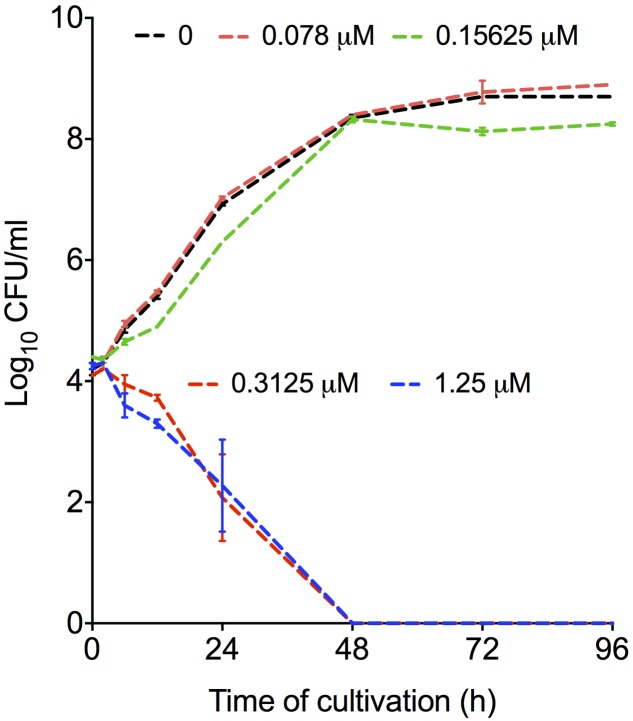
**Fungicidal activity of mebendazole against *C. neoformans.* Fungal cells were exposed to mebendazole (0.078–1.25 μM) for periods varying from 0 to 96 h.** Fungicidal activity was evident after 48 h and required a minimum concentration of 0.3125 μM mebendazole. In comparison to control systems (no drug), mebendazole concentrations of 0.3125 and 1.25 μM significantly affected fungal growth in all incubation periods. All the other drug concentrations resulted in fungal growth that was similar to that observed in the absence of mebendazole. Data illustrate a representative experiment of three independent replicates.

To test the fungicidal activity of mebendazole, *C. neoformans* was exposed to different drug concentrations for different periods of time. Mebendazole concentrations lower than 0.3125 μM had no effects on *C. neoformans* (**Figure 3**). At 0.3125 μM or higher, however, significant antifungal activity was observed after 6 to 12 h of exposure of *C. neoformans* to the drug. After 48 h, mebendazole killed 100% of *C. neoformans* cells.

### Activity of Mebendazole against Intracellular *C. neoformans*

**FIGURE 4:**
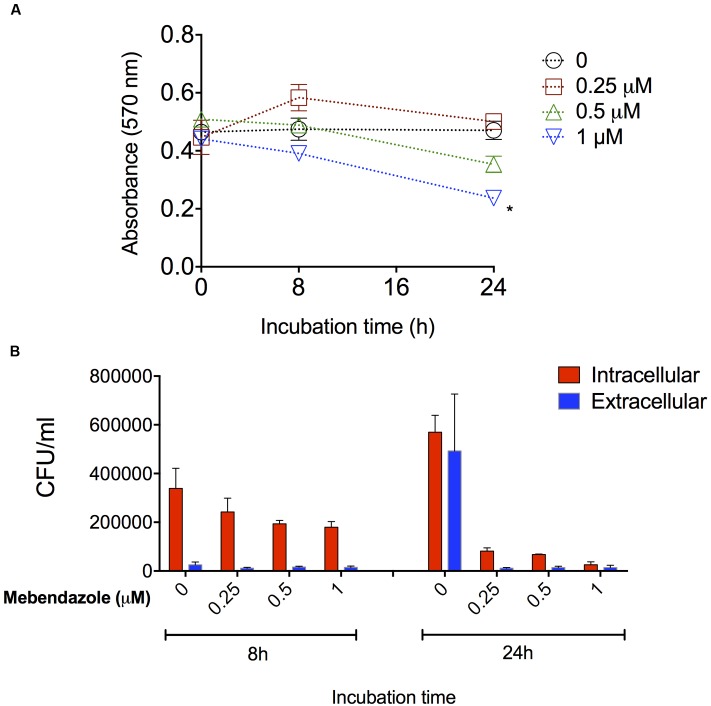
**Activity of mebendazole against intracellular *C. neoformans*.** **(A)** Viability of drug-treated, non-infected macrophages. After 8 h, all systems had similar viability levels (*P* > 0.1). After 24 h, cell viability was only affected by 1 μM mebendazole concentration (*P* = 0.0071). **(B)** Activity of mebendazole against intracellular (red bars) or extracellular (blue bars) *C. neoformans*. In intracellular assays, statistical differences (*P* < 0.05) between no drug (0) and drug-treated systems were always observed, except when the 0.25 μM concentration of mebendazole was used in the 8 h incubation period. In extracellular assays, statistical differences (*P* < 0.05) between no drug (0) and drug-treated systems were observed for all mebendazole concentrations, but only after the 24 h incubation. Comparative analysis of fungal loads obtained from intracellular and extracellular assays revealed statistical differences (*P* < 0.05) only in the 8 h period of incubation, suggesting that mebendazole is initially more effective against extracellular fungi, but similarly active against both intracellular and extracellular cryptococci after prolonged (24 h) periods of exposure to infected macrophages. Data illustrate a representative experiment of three independent replicates.

*Cryptococcus neoformans* is a facultative intracellular pathogen and this characteristic likely has a negative impact on the anti-cryptococcal treatment (Feldmesser et al., 2000). We then evaluated the ability of mebendazole to kill intracellular fungi. J774.16 macrophages were first infected with *C. neoformans* and then the cultures were treated with mebendazole for different periods (8 and 24 h) at variable drug concentrations (1, 0.5, and 0.25 μM). Macrophage viability was monitored by treating non-infected J774.16 cells with mebendazole alone (**Figure 4A**). After 8 h, no differences were observed between the viability of untreated macrophages and mebendazole-treated cells (*P* > 0.1). After 24 h, macrophage viability was affected by higher mebendazole concentrations (*P* = 0.0071 for 1 μM mebendazole), but no differences were observed between untreated phagocytes and cells that were exposed to 0.25 μM mebendazole (*P* = 0.3225). In infected macrophages, all mebendazole concentrations showed antifungal activity against both extracellular and intracellular fungi (**Figure 4B**). The concentration showing the lowest impact on macrophage viability (0.25 μM) was efficient in killing both intracellular and extracellular *C. neoformans.*

### Analysis of Potential Synergism between Mebendazole and Standard Antifungals

To evaluate whether the association of mebendazole with amphotericin B or fluconazole results in improved anti-cryptococcal activity, checkerboard assays were performed for calculation of the FIC index (**Table 1**). We found that mebendazole had additive activity against *C. neoformans* when combined with amphotericin B. Fluconazole did not show any improved effect in combination with mebendazole.

**Table 1:** Impact of the association of mebendazole with amphotericin B (AmB) or fluconazole (FLC) on antifungal activity.

Drug A			Drug B			FIC^∗^
		
	MIC Alone (μg/ml)	MIC Combined (μg/ml)		MIC Alone (μg/ml)	MIC Combined (μg/ml)	
Mebendazole	0.095	0.0475	AmB	0.25	0.125	1
Mebendazole	0.095	0.0475	Fluconazole	2	2	1.5


*^∗^Fractional inhibitory concentration index (FIC); Strongly synergistic: FIC < 0.5; Synergistic: FIC < 1; Additive: FIC = 1; No effect: 1 < FIC < 2; Antagonistic: FIC > 2.*

### Mebendazole Affects Capsule Size and Fungal Morphology

**FIGURE 5:**
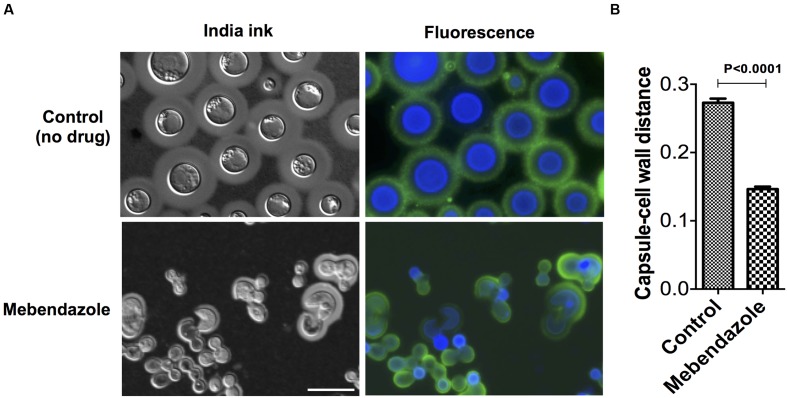
**Morphological alterations of *C. neoformans* cells treated with mebendazole.** **(A)** India ink counterstaining (left panels) and fluorescence microscopy (right panels) showing GXM (green fluorescence) and chitin (blue fluorescence) detection. Scale bar represents 10 μm. **(B)** Capsule measurement of India ink counterstained cells showing reduction in capsular dimensions by mebendazole. Data illustrate a representative experiment of three independent replicates.

To evaluate whether mebendazole affected key cellular structures of *C. neoformans* during regular growth, we cultivated the fungus for 48 h in the presence of a sub-inhibitory concentration (IC50; 0.22 μM) of the drug for further analysis of morphology and capsule size by a combination of fluorescence microscopy and India ink counterstaining. Fungal cells cultivated in the presence of mebendazole presented marked morphological alterations, including loss of spherical shape, intracellular furrows (**Figure 5A**) and reduced capsular dimensions (**Figure 5B**, *P* < 0.05).

### Effect of Mebendazole on GXM Release

**FIGURE 6:**
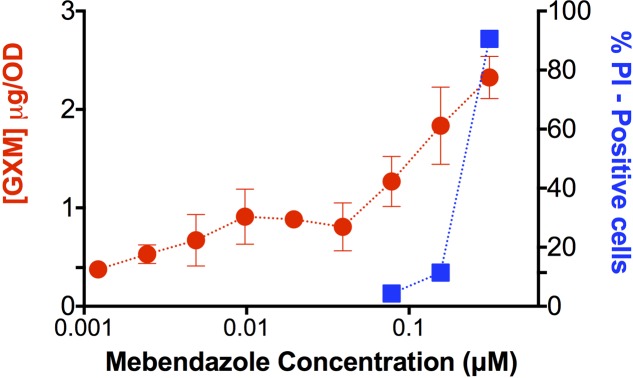
**Extracellular release of GXM by *C. neoformans* cells treated with different concentrations of mebendazole.** GXM extracellular detection correlated with increasing mebendazole concentrations. Staining of fungal cells with PI after exposure to fungicidal doses of mebendazole revealed a similar profile, suggesting polysaccharide leakage. Data illustrate a representative experiment of three independent replicates.

Since mebendazole interfered with capsule size (**Figure 5B**), we asked whether GXM release was affected by exposure of *C. neoformans* to the drug. Supernatants of fungal cells cultivated in the presence of mebendazole were used for GXM quantification by ELISA (**Figure 6**). Unexpectedly, supernatants of mebendazole-treated cells had increased GXM concentration, mainly in the dose range required for fungal killing. Since the polysaccharide is synthesized intracellularly (Yoneda and Doering, 2006), we hypothesized that the increased GXM detection would result from leakage induced by membrane damage. To address this possibility, mebendazole-treated cells were stained with PI. Stained cells varied from 70 to 90% in the dose range generating cell death, which was compatible with membrane damage and polysaccharide leakage. Noteworthy, these results and those described in **Figure 5** are also compatible with the hypothesis of GXM release from the cell surface, so we cannot rule out the possibility that mebendazole promotes detachment of capsular structures in *C. neoformans*.

### Activity of Mebendazole against *C. neoformans* Biofilms

**FIGURE 7:**
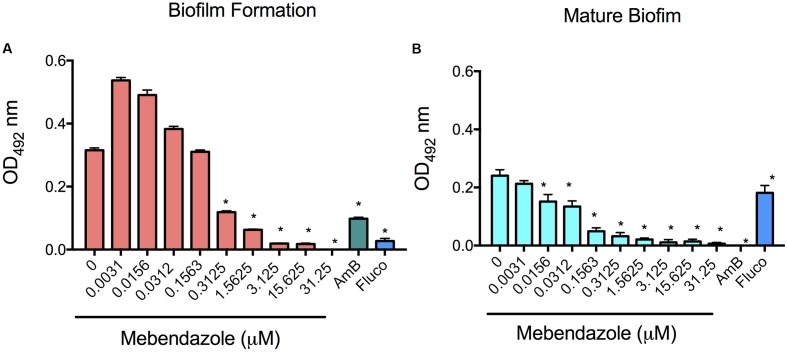
**Effects of mebendazole on cryptococcal biofilms.** Biofilm formation and mature biofilm activity were measured indirectly by XTT reduction assay. **(A)** Incubation of *C. neformans* with mebendazole before biofilm formation. **(B)** Treatment of mature biofilms with mebendazole. AmB, amphotericin B (2 μg/ml); Fluco, fluconazole (8 μg/ml). Statistical significances after comparison with control systems are indicated by asterisks (*P* < 0.0001). Data illustrate a representative experiment of three independent replicates.

Biofilm formation causes well-known difficulties in the treatment of a number of infectious diseases, including cryptococcosis (Martinez and Casadevall, 2006; Borghi et al., 2016). Based on this observation, we evaluated whether co-incubation of mebendazole with yeast cells prevented *C. neoformans* biofilm formation or caused damage to mature biofilms. The metabolic activity was measured by XTT reduction assay (**Figure 7**). Fluconazole is known to have no effects on mature biofilms, in contrast to amphotericin B (Martinez and Casadevall, 2006). Therefore, these drugs were used as negative and positive controls, respectively. Mebendazole at MIC (0.3125 μM) affected biofilm formation (**Figure 7A**; *P* < 0.0001) and damaged mature biofilms (**Figure 7B**; *P* < 0.0001). Lower concentrations of mebendazole similarly affected *C. neoformans* mature biofilms (**Figure 7B**; *P* < 0.0001). As expected, higher concentrations of mebendazole had even clearer impacts on *C. neoformans* biofilms.

### Identification of Potential Cellular Targets for Mebendazole

**FIGURE 8:**
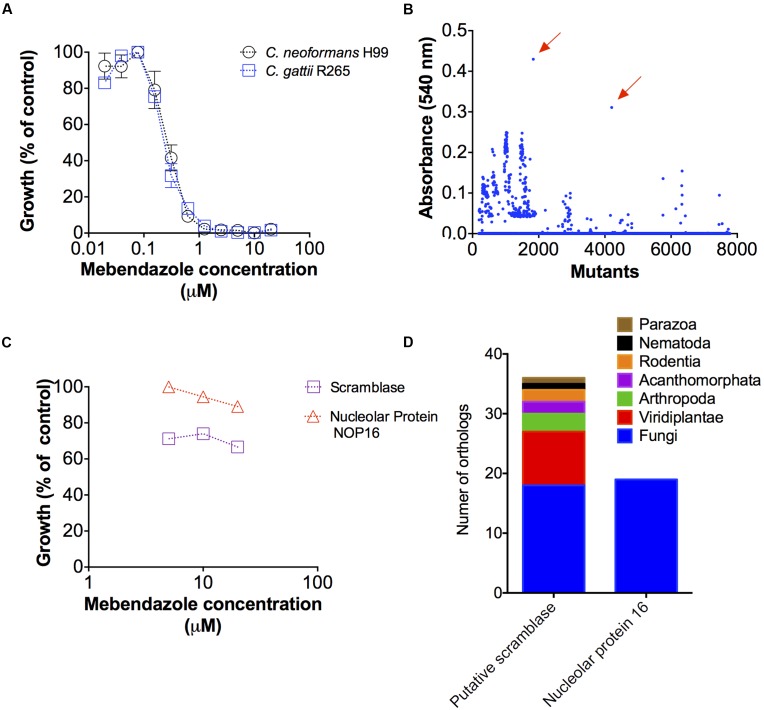
**Potential cellular targets required for mebendazole activity.** **(A)** Comparative analysis showing that *C. neoformans* and *C. gattii* are similarly susceptible to mebendazole. **(B)** Screening of a *C. gattii* mutant library in the presence of 10 μM of mebendazole, revealing two highly resistant strains (arrows). **(C)** Growth of the two mutants selected in panel B in the presence of variable concentrations of mebendazole. **(D)** Sequence identification by PCR and distribution of cellular proteins required for mebendazole activity.

Antifungal activities of benzimidazoles were described before (Cruz et al., 1994), but the mechanisms by which these drugs affected cryptococcal growth remained unknown. In order to identify potential targets for mebendazole in *Cryptococcus* spp., we first evaluated whether the drug affected the growth of *C. gattii* and *C. neoformans* in a similar fashion. In fact, growth inhibition curves were identical for both pathogens (**Figure 8A**).

A mutant collection generated by co-incubation of *C. gattii* and *A. tumefaciens* (Idnurm et al., 2004) was produced by our group for general purposes involving development of antifungals and pathogenic studies. In the present study, these mutants were screened for resistance phenotypes in the presence of 10 μM mebendazole, based on the assumption that, in the absence of a cellular target required for antifungal activity, the drug would lack anti-cryptococcal properties. Most of the mutants were sensitive to mebendazole and a number of strains were partially resistant to the drug (**Figure 8B**). However, two of the mutant strains clearly stood out from the entire collection, showing highest levels of resistance even in higher mebendazole concentrations (**Figure 8C**). Interrupted regions in these two mutants were identified by inverse PCR (**Figure 8D**). In the most mebendazole-resistant mutant, the protein codified by the interrupted gene contained a scramblase domain (PF03803 – CNBG_3981). In the second most resistant mutant, the gene coding for ribosome biogenesis protein Nop16 (PF09420 – CNBG_3695) was interrupted. These domains were found in diverse phylogenetic groups, according to the PFAM database (Finn et al., 2016). However, orthologs for these genes were found in a narrow group of organisms and absent in human cells (**Figure 8D**), according to OrthoMCL database (Chen et al., 2006). These results indicate that at least two novel cellular targets are involved in the antifungal activity of mebendazole.

**FIGURE 9:**
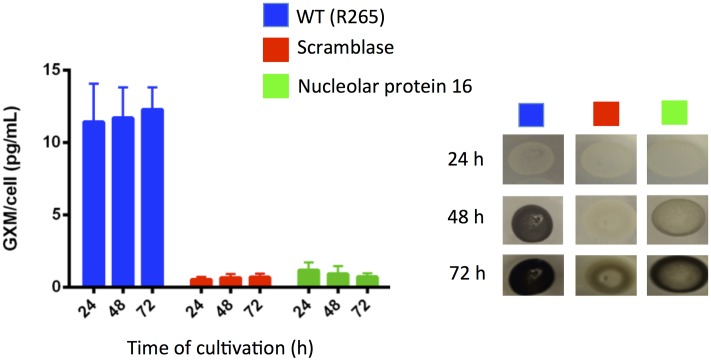
***Cryptococcus gattii* mutants lacking potential targets for mebendazole activity show defective capacity to produce well-known cryptococcal virulence factors.** GXM determination by ELISA (left panel) indicated that the putative scramblase and nucleolar protein 16 were required for polysaccharide export. Both proteins were apparently involved in the kinetics of pigmentation in *C. gattii* (right panel).

Neutralizing cryptococcal virulence factors is also likely to be beneficial for the control of cryptococcosis. In this context, we evaluated whether the mutants lacking potential targets for activity of mebendazole had normal production of the most-well characterized cryptococcal virulence factors. The two mutants had normal urease activity (not shown). Analysis of extracellular GXM and pigmentation, however, showed important differences between WT and mutant cells (**Figure 9**). Mutants disrupted for expression of the putative scramblase and of nucleolar protein 16 had decreased contents of extracellular GXM, in comparison with WT cells (*P* < 0.001). The kinetics of melanin production was also negatively affected in the mutants.

## Discussion

Processes of drug research and development are costly, time consuming and have questionable success (Kaitin, 2010; Kaitin and DiMasi, 2011). In this scenario, drug repurposing has provided a potential boost to the drug pipeline combating health emergencies and assisting neglected populations. Recent studies have provided evidence of successful drug repurposing to combat *Cryptococcus* (ClinicalTrials.gov, 2016; Rhein et al., 2016) and Zika virus (Veljkovic and Paessler, 2016; Xu et al., 2016; Sacramento et al., 2017) infections with promising results. The need for additional anti-infectious agents, however, is clear.

In this study, we identified small-molecule inhibitors of *C. neoformans* via a drug-repurposing screen. Our findings demonstrated antifungal activity in a group of anti-helminthic benzimidazoles and suggested potential targets for development of novel antifungals. Benzimidazoles are heterocyclic aromatic bis-nitrogen azoles that are considered promising anchors for development of new therapeutic agents (Bansal and Silakari, 2012). Benzimidazole derivatives have been associated to the control of infectious diseases through antiviral, antifungal, antimicrobial, and antiprotozoal properties, but they also manifest antiinflammatory, anticancer, antioxidant, anticoagulant, antidiabetic and antihypertensive activities (Ates-Alagoz, 2016). The anti-cryptococcal activity of benzimidazoles was demonstrated two decades ago (Cruz et al., 1994), but the effects of these compounds on fungal morphology and biofilm formation were not explored. Similarly, their cellular targets and ability to kill intracellular fungi remained unknown. Mebendazole, one of the benzimidazoles showing antifungal activity, is in clinical trial for the treatment of human pediatric glioma (ClinicalTrials.gov, 2013). Since effective anti-cryptococcal agents obligatorily need to reach the central nervous system at biologically active concentrations, we selected mebendazole for our experiments of antifungal activity. This compound affected cryptococcal growth, morphology, biofilms and macrophage infection.

The pathogenic mechanisms used by *C. neoformans* during infection bring significant complexities in the management of cryptococcosis. Conditions favoring biofilm formation are thought to contribute to cryptococcal virulence (Benaducci et al., 2016). Like with other pathogens, *C. neoformans* biofilms are resistant to antimicrobial agents and host defense mechanisms, causing significant morbidity and mortality (Martinez and Casadevall, 2015). These characteristics are especially relevant in a scenario of increasing use of ventriculoperitoneal shunts to manage intracranial hypertension associated with cryptococcal meningoencephalitis (Martinez and Casadevall, 2015). In our model, mebendazole was an efficient antifungal agent against *C. neoformans* biofilms. The minimum mebendazole concentration required for antifungal activity against planktonic cells (0.325 μM) was much higher than the doses required for activity against mature biofilms (0.0156–0.0312 μM). The reason for this discrepancy is unclear. Since capsular polysaccharides have well described roles on the assembly of cryptococcal biofilms (Martinez and Casadevall, 2005), we hypothesize that the herein described impact of mebendazole on capsular architecture and GXM release may affect biofilm stability.

An additional complexity in the treatment of cryptococcosis is the ability of the fungus to reside inside phagocytes. In fact, persistent pulmonary infection is associated with the intracellular parasitism of *C. neoformans* (Goldman et al., 2000). In this context, targeting intracellular survival and growth and/or cryptococcal virulence factors expressed during intracellular parasitism might offer new strategies to improve anticryptococcal treatment, as reviewed by Voelz and May (2010). Mebendazole was effective against phagocytized fungi in our model. The fact that the anti-helminthic compound had an additive effect to AmB also suggests that benzimidazole-like compounds could be used in therapeutic protocols against cryptococcosis.

The primary mechanism of anthelmintic activity of mebendazole relies on binding the β-subunit of tubulin before dimerization with α-tubulin, with subsequent blocking of microtubule formation (Gardner and Hill, 2001). Tubulin may also be the target of mebendazole in *C. neoformans*, but our studies suggest the possibility that the antifungal effects of mebendazole may involve additional targets. Mebendazole induced membrane permeabilization, as concluded from increased levels of PI staining after exposure of *C. neoformans* to the drug. In addition, mutants lacking genes coding for a putative scramblase and the nucleolar protein Nop16 were highly resistant to mebendazole. Both mutants had defective formation of important virulence factors. Remarkably, sequences showing similarity to these two proteins were absent in human cells, suggesting a great potential for these two proteins as novel antifungal specific targets. Scramblases are ATP-independent enzymes that act to randomize lipid distribution by bidirectionally translocating lipids between leaflets (Hankins et al., 2015). Lipid-translocating enzymes, in fact, are fundamental for cryptococcal pathogenesis and GXM export (Hu and Kronstad, 2010; Rizzo et al., 2014). The functions of Nop16 in *C. neoformans* are unknown, but in *S. cerevisiae* this protein is a component of 66S pre-ribosomal particles required for 60S ribosomal subunit biogenesis (Harnpicharnchai et al., 2001; Horsey et al., 2004). Although there is no evidence in the literature linking tubulin polymerization, membrane permeability and cellular functions of these two potential targets, our studies suggest that a connection may exist and these proteins may be functionally integrated in fungi.

Cryptococcosis affects regions where health infrastructure resources are extremely limited. Considering the high mortality rates associated with this disease and the socio-economic scenario behind cryptococcosis, low-cost and efficient antifungal alternatives are urgent. Clinical use of novel drugs, however, depends on a number of properties of the molecular candidates. In this regard, the potential use of mebendazole against human cryptococcosis raises several concerns. For instance, the use of mebendazole at large doses may cause bone marrow suppression (Fernandez-Banares et al., 1986) and it is unclear if the compound is safe in pregnancy (Torp-Pedersen et al., 2012). Benzimidazoles have only limited water solubility, which impacts the rate and extent of their absorption and, consequently, systemic bioavailability, maximal plasma concentration, and tissue distribution (McKellar and Scott, 1990). Animal studies with mebendazole demonstrated drug distribution through all the organs, including the central nervous system (Lanusse and Prichard, 1993). However, the drug and its metabolites were concentrated mainly in the liver, where they remain for at least 15 days post treatment (Lanusse and Prichard, 1993). Mebendazole is thought to be the active form of the drug rather than its metabolites (Gottschall et al., 1990). However, in the liver, benzimidazoles are mostly modified by the enzymatic system of hepatic microsomal oxidases, which are involved in sulfoxidation, demethylation, and hydroxylation (Short et al., 1988; Lanusse and Prichard, 1993). In fact, benzimidazoles are usually short lived and metabolic products predominate in plasma and all tissues and excreta of the host (Fetterer and Rew, 1984).

Although the difficulties associated with the clinical use of mebendazole are clear, our results combine a multiple antifungal activity with molecular targets that are absent in human cells, which encourages further development of benzimidazole-like molecules against *C. neoformans*. In fact, a number of reports have suggested that the benzimidazole core represents a promising scaffold for development of new therapeutic agents (Yadav and Ganguly, 2015). Montresor et al. (2010) estimated that the cost to procure one million doses of standard benzimidazoles (500 mg each) would be approximately US$ 20,000, including international transport. In this context, the ability of mebendazole to penetrate the brain (Bai et al., 2015) and to cause expressive damage in *C. neoformans* cells suggest great potential as a prototype for development of novel anti-cryptococcal agents.

## Author Contributions

LJ, CS, LK, AS, MDP, MV, and MR prepared the experimental design. LJ, RS, WL, RA, and CS performed the experiments. LJ, CS, LK, AS, MDP, MV, and MR discussed the results, wrote and approved the final manuscript.

## Conflict of Interest Statement

The authors declare that the research was conducted in the absence of any commercial or financial relationships that could be construed as a potential conflict of interest. Part of the data presented here is also the subject of a pending patent application (Ref.: BR1020170047300, National Institute of Industrial Property - INPI, Brazil). The reviewer JNDAJ and handling Editor declared their shared affiliation, and the handling Editor states that the process nevertheless met the standards of a fair and objective review.

## Footnotes

1 http://www.biomol.unb.br/phph/

2 http://www.broadinstitute.org/scientific-community/science/projects/fungal-genome-initiative/cryptococcus-gattii-genome-project-0
